# Increased Relative Abundance of *Ruminoccocus* Is Associated With Reduced Cardiovascular Risk in an Obese Population

**DOI:** 10.3389/fnut.2022.849005

**Published:** 2022-04-28

**Authors:** Arun Prasath Lakshmanan, Sara Al Zaidan, Dhinoth Kumar Bangarusamy, Sahar Al-Shamari, Wahiba Elhag, Annalisa Terranegra

**Affiliations:** ^1^Precision Nutrition, Research Department, Sidra Medicine, Doha, Qatar; ^2^Bariatric and Metabolic Surgery Department, Hamad Medical Corporation, Doha, Qatar

**Keywords:** *Ruminococcus*, CVD risk, obesity, vitamin D, diet

## Abstract

**Background:**

Obesity is a complex disease with underlying genetic, environmental, psychological, physiological, medical, and epigenetic factors. Obesity can cause various disorders, including cardiovascular diseases (CVDs), that are among the most prevalent chronic conditions in Qatar. Recent studies have highlighted the significant roles of the gut microbiome in improving the pathology of various diseases, including obesity. Thus, in this study, we aimed to investigate the effects of dietary intake and gut microbial composition in modulating the risk of CVD development in obese Qatari adults.

**Methods:**

We enrolled 46 adult subjects (18–65 years of age) who were classified based on their CVD risk scores, calculated using the Framingham formula, into a CVD no-risk group (score of <10%, *n* = 36) and CVD risk group (score of ≥10%, *n* = 10). For each study subject, we measured the gut microbial composition with a 16s rDNA sequencing method that targeted the v3-v4 region using Illumina Miseq, and their nutritional status was recorded based on 24-h dietary recall. Dietary intake, bacterial taxa summary, diversity index, microbial markers, pathway analysis, and network correlation were determined for the study subjects.

**Results:**

The CVD risk group showed a lower intake of vitamin D, reduced relative abundance of genera *Ruminococcus* and *Bifidobacterium*, no change in bacterial diversity, and higher levels of taurine, hypotaurine, and lipoic acid metabolism than the CVD no-risk group. Besides, the relative abundance of genus *Ruminococcus* was positively correlated with the intake of protein, monounsaturated fat, vitamin A, and vitamin D.

**Conclusion:**

Taken together, our results suggest that the genus *Ruminococcus* could be used as a microbial marker, and its reduced relative abundance could mediate the risk of CVDs in the Obese Qatari population.

## Introduction

According to a recent WHO report, the prevalence of obesity has reached pandemic proportions, and the obesity-related mortality rate is at least 2.8 million people each year worldwide. A recent cross-sectional study conducted on Qatari adults suggested that the prevalence rate for obesity is 33% (1), and the rate is higher in women compared to men. Obesity is a complex network of pathologies, as it is a multifactorial disorder that includes genetic, environmental, psychological, physiological, medical, and epigenetic factors (2–5), and its underlying mechanisms mainly include (1) an imbalance in the ratio between the accumulation of excess fat and energy expenditure by the body and (2) resetting of the set bodyweight point to a higher value (2, 6). In addition to these factors, the gut microbiota has been shown to play an unprecedented role in the pathogenesis of obesity; crosstalk between the gut microbiota and obesity is like a double-edged sword because both entities are regulated by each other (7–9).

Obesity is also closely associated with the pathogenesis of cardiovascular diseases (CVDs), including cardiac arrhythmias, congenital heart disease, coronary heart disease, heart failure, cardiomyopathy, and pulmonary embolism (10, 11). A prominent pathway linked to obesity-associated CVDs is the activation of inflammatory mediated pathways, for example, the secretion of lipopolysaccharide synthesis, which mainly acts through toll-like receptors, causes the aggravation of atherosclerotic plaque formation (12). Furthermore, a handful of evidence from clinical and pre-clinical studies has shown that the gut microbiota also plays a crucial role in obesity-associated CVDs, primarily through the secretion of gut microbiota-derived metabolites such as L-carnitine, betaine, choline, TMAO, and short-chain fatty acids (SCFAs) (13–15), and the fat content of the diet influences how the gut microbiota effects CVDs (8, 16, 17). Marzullo et al. (18) demonstrated that obesity is associated with CVDs through its modulation of the gut microbiota at both lower taxonomic and higher taxonomy levels of change. Gut microbial diversity is an important indicator of the health status of the gut: the higher the diversity, the healthier the gut. Pre-clinical and clinical studies have demonstrated that microbial diversity decreases with the severity of obesity (8, 17, 19, 20). On the other hand, Lin et al. (21) studied the microbial diversity in Chinese subjects and found no relationship between microbial diversity and body mass index (BMI). Obesity has been demonstrated to increase CVD risk, mainly through gut microbial dysbiosis, which can cause a leaky gut and lead to CVDs through a variety of mechanisms, such as secretion of SCFAs and TMAO, potentiation of systemic inflammation, and regulation of G-protein receptors (GPRs) (18). In addition, Fu et al. demonstrated that the gut microbiota contributes to variations in lipid profiles, especially triglycerides and HDLs, independently of BMI, and these changes are small but contribute significantly to outcomes (22).

Numerous studies have demonstrated that diet is a predominant factor in the modulation of gut microbiota (23, 24), and it can even abruptly change the gut microbiota on a daily timescale in humans (25), suggesting its potential role in maintaining the health of the gut microbiota. Multiple studies have demonstrated that the diet is one of the most prominent epigenetic factors affecting the gut microbial composition in terms of both richness and abundance (23, 24). It is believed that acute dietary adjustments can change the microbial composition substantially, but because of the stability of the enterotype, they do not cause a switch from one enterotype to another; whereas chronic dietary adjustments can lead to the formation of a different enterotype (26).

Thus, in this study, we aimed to characterize the gut microbial composition and search for links between dietary patterns, gut microbial dysbiosis, and the development of CVDs in Qatari obese adults. We also aimed to explore the molecular pathway mechanisms that may contribute to the development of obesity-associated CVDs and that involve the gut microbiota in this population.

## Patients and Methods/Materials

### Recruitment of Study Participants

The study participants were approached and recruited after having consented to participation at Hamad Medical Corporation, Doha, Qatar. This study was approved by the Institutional Review Boards of both Sidra Medicine (IRB, #1604002867) and Hamad Medical Corporation (IRB, #16419/16). We applied two inclusion criteria: (1) Qatari citizens with BMI > 30 kg/m^2^ and (2) male or female in the age range of 18–65 years. The exclusion criteria were those who had had (1) bariatric surgery, (2) chronic diseases other than obesity, and (3) antibiotics in the last 3 months. A total of 46 study participants were included. From each study participant, we collected stool and blood samples, and dietary data were collected by a trained dietician, who interviewed the patients on their 24-h dietary recall. Anthropometric measurements, such as body weight, height, and waist circumference, as well as blood pressure (BP) and heart rate (HR) were measured.

### Nutrient Intake

The 24-h food recall data were collected by the dietician and computed using Nutritionist Pro software (Axxya Systems LLC) to obtain the micro- and macro-nutrient intake for each study participant. The obtained data were used to compare the CVD risk and CVD no-risk groups.

### Biochemical Parameters

Blood samples were used to measure various biochemical parameters, such as fasting blood glucose (FBG), glycosylated hemoglobin (HbA1c), insulin, lipid profiles (HDL, LDL, and TG), alkaline phosphatase (ALP), alanine transaminase (ALT), aspartate transaminase (AST), thyroid stimulated hormone (TSH), triiodothyronine (T3), thyroxine (T4), vitamin D, vitamin B12, folate, uric acid, and iron saturation levels.

### Calculation of CVD Risk Score

The CVD risk score was calculated using the Framingham risk score method (27), mainly considering age, gender, smoking habits, systolic blood pressure, total cholesterol and HDL levels, HbA1C, and treatment for hypertension.

### 16s rDNA Sequencing

Stool samples were collected in an OMNI-gene Gut tube, and microbial genomic DNA (gDNA) was extracted using QIAamp Fast DNA Stool Mini Kit (Qiagen, catalog number #51604). The extracted gDNA was amplified using primers that targeted the v3-v4 region of 16s rDNA, indexed using Nextera XT kit v2 (catalog number #FC-131-1002, Illumina), and sequenced according to the Illumina protocol described previously (28) using MiSeq Reagent Kit v3 for 600 cycles (catalog number #MS-102-3003, Illumina).

### Gut Microbial Computational Analysis

#### Classification of Taxa

The raw fastq files were demultiplexed using MiSeq Control Software. To merge the forward and reverse sequences, we used a paired-End read mergeR v0.9.8 tool (29), and the merged reads were trimmed using the Trimmomatic v0.36 tool (30) to obtain reads with a quality score of >30. The Quantitative Insights Into Microbial Ecology (QIIME) v1.9.0 pipeline (31) was used to convert the FASTQ files into FASTA files, which were all combined into a single FASTA file. The Greengenes database (gg_13_08) was used to obtain the operational taxonomic units (OTUs) by aligning the sequences with a confidence threshold of 97% (32).

#### Gut Microbial Diversity Indices

Alpha diversity is a measure of microbial richness and relative abundance, and it was estimated by R packages (such as Phyloseq and ggplot2), using the Observed, Chao1, Shannon, and Simpson methods, as previously described (28). Beta diversity is a measure of the similarity or dissimilarity of the microbial composition between groups, and it was determined by principal coordinate analysis as proposed in QIIME v1.9.0 (Bray-Curtis method) and as previously described (28).

#### Identification of Gut Microbial Markers

Gut microbial markers were estimated using the linear discriminant analysis effect size (LEfSe) tool as described previously (33). The LEfSe tool uses a non-parametric factorial Kruskal–Wallis rank sum test to identify features with significantly differential relative abundance, followed by Linear Discriminant Analysis (LDA) to calculate the effect size of each differentially abundant microbial feature. Features were considered significant if the LDA value was >2.0.

#### Functional Pathway Profiling

We performed Phylogenetic Investigation of Communities by Reconstruction of Unobserved States (PICRUSt) analysis according to the literature review by Langille et al. (34). PICRUSt is a bioinformatics software package designed to predict metagenome functional content from marker gene surveys.

#### Association of Marker Microbes and Host Phenotype

We used a Statistical Inference of Associations between Microbial Communities and host phenoTypes (SIAMCAT) machine learning tool to identify the associations between marker microbes and host phenotype, and SIAMCAT analysis was performed according to Wirbel et al. and following ridge logistic regression analysis as described previously (35).

#### Correlation Between Diet and Microbial Composition

Correlation network analysis between diet and microbial composition was performed using GraphPad Prism software.

#### Statistical Analysis

A normality test was used to check the distribution of the patients' phenotypic data. Unless otherwise specified, data are presented as the median and interquartile range (IQR). Comparisons of the two groups were performed with Student's *t*-test and Mann–Whitney test, wherever applicable on Prism Software version 8, (GraphPad). A value of *p* < 0.05 was considered statistically significant.

## Results

### Demographic, Anthropometric, Clinical, and Biochemical Parameters of Study Participants

First, we estimated the demographic, anthropometric, clinical, and biochemical parameters for each study participant. Interestingly, we did find statistically significant difference in age, but not HR parameter between the CVD no-risk and CVD risk groups (Table 1). Additionally, we measured diabetes mellitus-related parameters, lipid profiles, liver function profiles, vitamins, thyroid function profiles, uric acid, and iron saturation levels for each patient. The CVD risk group was found to have higher levels of FBG, HbA1c, ALT, AST, and vitamin B12 than the CVD no-risk group (Table 1). The actual number of subjects used to derive the above data is mentioned in Supplementary Table 1.

**Table 1:** Demographic, clinical, and biochemical parameters of the study participants.

	**CVD no-risk**	**CVD risk**	***P*-value (*t*-test)**
**Number of Study participants**	36	10	NA
Age (y)	41.8 ± 9.3	53.1 ± 8.3	0.0009
**Gender**			
Male	8	3	NA
Female	28	7	NA
**Glucose-related parameters**			
FBG in mmol/L	5.1 (4.8–5.7)^†^	6.4 (5.8–8.3)	0.0003
HbA1c in %	5.5 (5.1–5.7)^†^	6.8 (6.1–8.0)	<0.0001
Insulin in IU	15.4 (9.8–25.3)^†^	15.8 (11.4–22.3)^†^	0.824^ns^
**Complete lipid profiles**			
TC in mmol/L	4.8 (4.3–5.4)^†^	5.0 (4.3–5.4)	0.946^ns^
TG in mmol/L	1.1 (0.9–1.5)^†^	1.5 (1.1–1.8)	0.181^ns^
HDL in mmol/L	1.3 (1.1–1.5)^†^	1.3 (1.0–1.5)	0.513^ns^
LDL in mmol/L	2.9 (2.5–3.7)^†^	2.7 (2.3–3.7)	0.794^ns^
**Liver function parameters**			
ALP in U/L	65.0 (56.0–81.0)^†^	73.0 (64.0–106.0)^†^	0.129^ns^
ALT in IU/L	20.0 (11.0–28.0)^†^	38.0 (35.5–45.5)^†^	0.0007
AST in U/L	17.0 (14.0–23.0)^†^	23.0 (19.5–27.5)^†^	0.014^ns^
**Thyroid function parameters**			
TSH in mIU/L	1.9 (1.4–3.2)^†^	1.7 (1.1–2.5)^†^	0.330^ns^
T3 pmol/L	4.3 (3.6–4.8)^†^	4.4 (3.8–5.1)^†^	0.731^ns^
T4 in pmol/L	15.0 (13.2–15.7)^†^	14.1 (12.4–16.2)^†^	0.618^ns^
**Vitamins**			
Folate (vitamin B9) in nmol/L	22.7 (16.0–34.8)^†^	23.3 (18.5–32.5)^†^	0.714^ns^
Vitamin D ng/mL	20.0 (12.0–27.5)^†^	20.5 (16.5–27.75)	0.605^ns^
Vitamin B12 pmol/L	258.0 (217.5–328.0)^†^	364.0 (274.3–496.3)	0.010
**Clinical parameters**			
HR	80.0 (72.0–89.5)^†^	80.5 (72.0–84.8)	0.635^ns^
Systolic BP	122.0 (112.0–131.0)^†^	130.5 (124.8–140.0)	0.028
Diastolic BP	73.5 (64.5–84.0)	77.0 (69.0–88.25)	0.381^ns^
**Others**			
Uric acid in mmol/L	278.0 (242.3–327.5)^†^	344.0 (264.0–440.8)^†^	0.109^ns^
Iron saturation level in %	16.0 (11.0–19.6)^†^	16.5 (12.2–24.8)	0.574^ns^

*All values are expressed as median and IQR. Student's t-test or Mann-Whitney test was performed, wherever applicable P <0.05 was considered statistically significant; ns, not significant. FBG, fasting blood glucose; HbA1c, glycosylated hemoglobin; TC, total cholesterol; TG, triglycerides; HDL, high-density lipoprotein; LDL, low-density lipoprotein; ALP, alkaline phosphatase; ALT, alanine transaminase; AST, aspartate transaminase; TSH, thyroid-stimulating hormone; T3, triiodothyronine; T4, thyroxine; HR, heart rate; BP, blood pressure; mmol/L, millimole per liter; U/L, units per liter; IU/L, international units per liter; ng/mL, nanogram per milliliter; mIU/L, milli international units per liter; pmol/L, picomole per liter*.

† *Indicates that the value was derived from less than the actual study participants (refer to Supplementary Table 1)*.

### Nutrient Intake of CVD No-Risk and CVD Risk Study Participants

**Figure 1:**
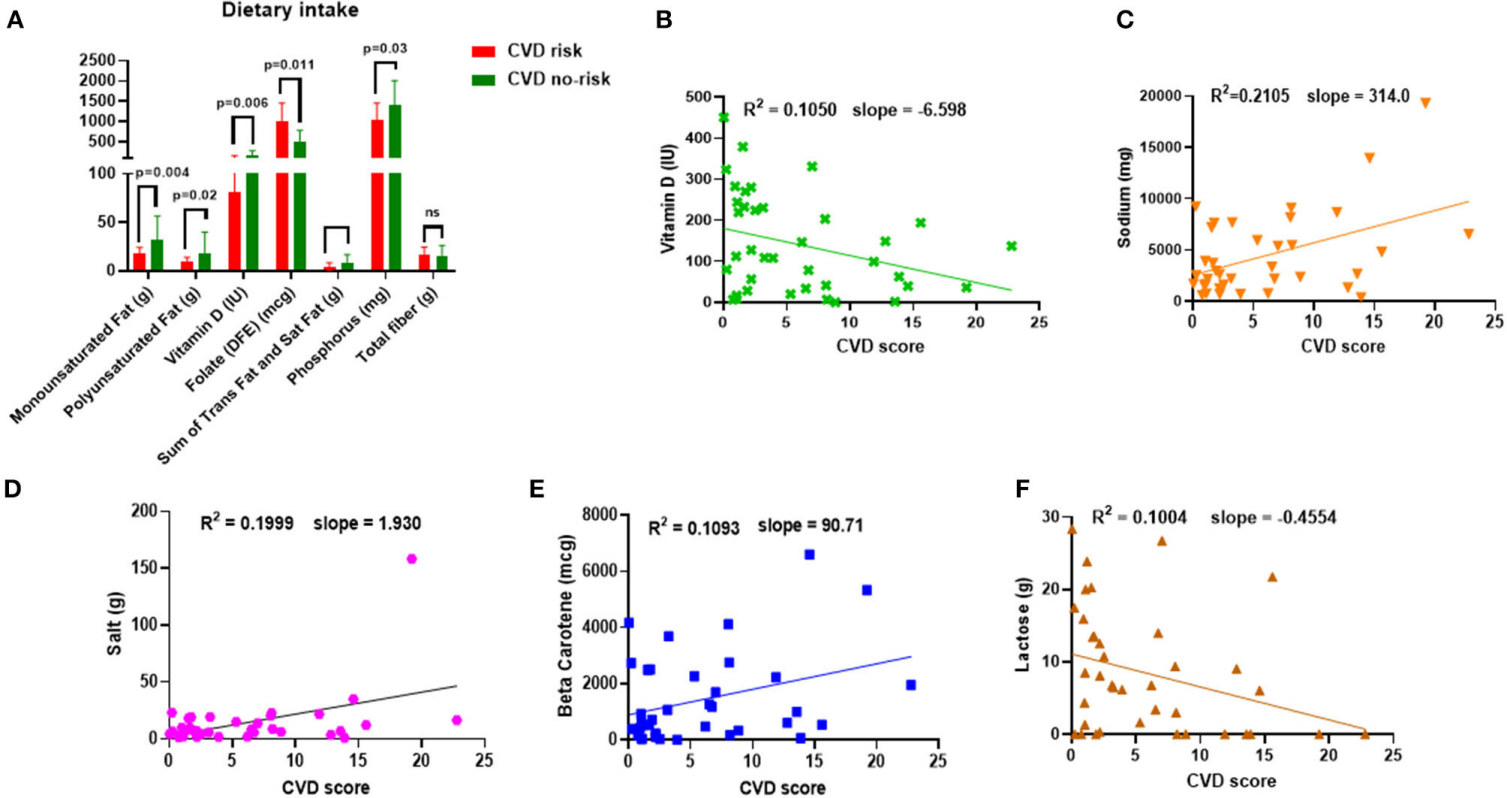
Dietary intake and correlation to CVD risk. **(A)** Dietary intake of MUFA, PUFA, vitamin D, folate, the sum of trans-fat and saturated fat, phosphorus, and total fiber in the CVD no-risk and CVD risk groups. **(B–F)** Correlation analysis of nutrient intake (sodium, salt, beta-carotene, lactose, and vitamin D) and CVD risk in the obese population. CVD no-risk *N* = 30; CVD risk *N* = 8. *P* < 0.05 was considered statistically significant using Student's *t-test* or Mann-Whitney test was performed, wherever applicable.

We calculated the macro-and micro-nutritional intake of each study participant. The consumption of MUFA, polyunsaturated fat (PUFA), vitamin D, the sum of trans-fat and saturated fat, and phosphorus was significantly lower, but interestingly, folate consumption was higher in the CVD risk group than in the CVD no-risk group (Figure 1A). In addition, we attempted to find a correlation between nutritional intake and the risk of CVD and found that dietary consumption of sodium, salt, and beta-carotene positively correlated with CVD risk score, whereas vitamin D and lactose were negatively correlated with CVD risk (Figures 1B–F). The significant results in both the comparison and correlation analyses of vitamin D intake suggest vitamin D has a crucial role in the development of obesity-associated CVDs (Figures 1A,B).

### Gut Microbial Composition in Obese Subjects With Increased CVD Risk

**Figure 2:**
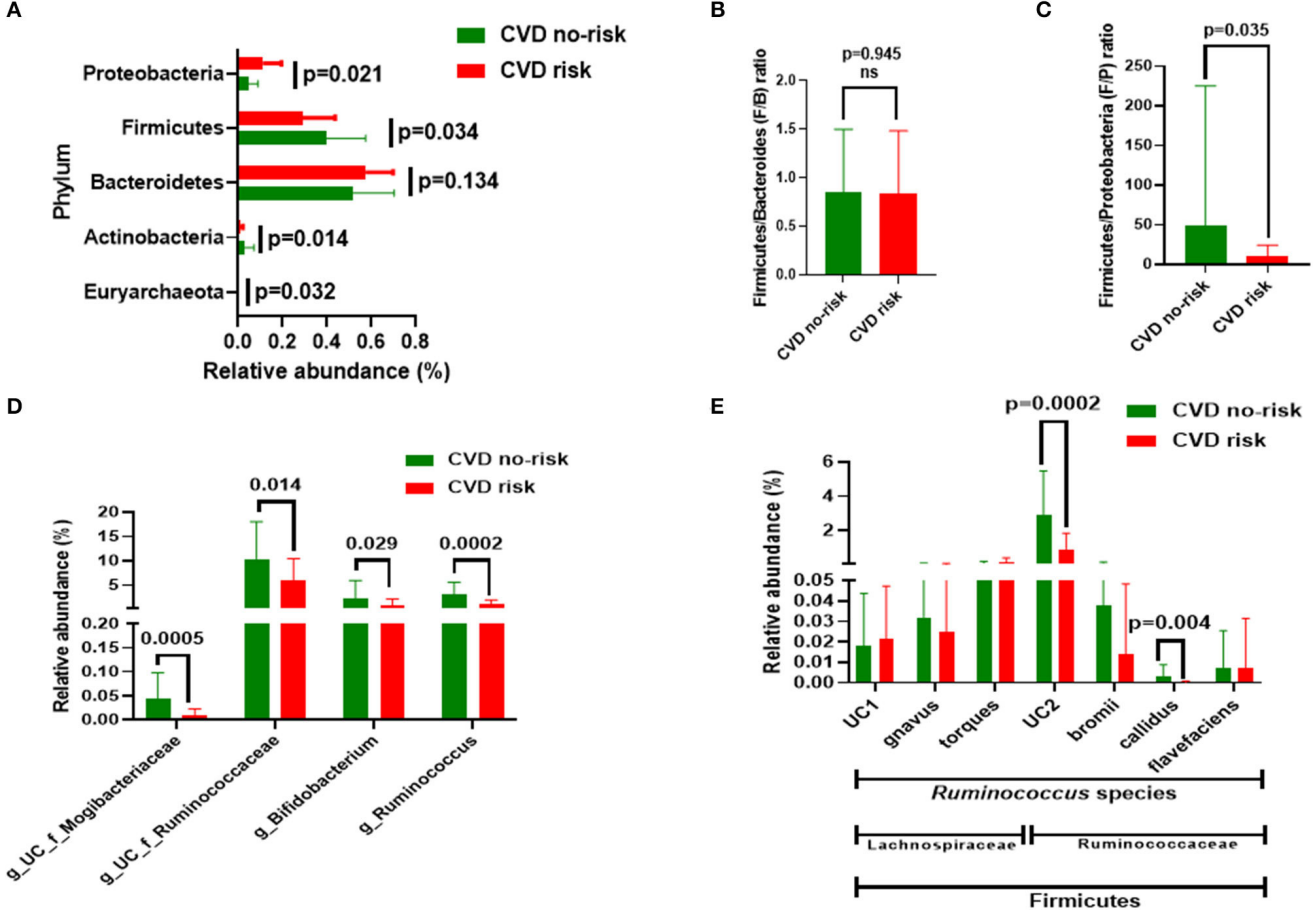
Gut microbial composition in the CVD no-risk and CVD risk groups. **(A)** The four major bacterial phyla, except Euryarchaeota, were found in both groups. Data are shown as relative abundance (percentage of total gut microbiota composed of each phylum). **(B,C)** Ratio of Firmicutes to Bacteroidetes and Firmicutes and Proteobacteria, respectively, in the CVD no-risk and CVD risk groups. **(D)** Relative abundance of four major genera in the CVD no-risk and CVD risk groups. **(E)** Relative abundance of species of the *Ruminococcus* genus from the families of Ruminococcaceae and Lachnospiraceae in the CVD risk and CVD no-risk groups. The results are expressed as mean ± SD. CVD no-risk, *N* = 36; CVD risk, *N* = 10. *P* < 0.05 was considered statistically significant using Student's *t-*test. “g_UC” and “f_UC” represent unclassified bacteria at the genus and family level, respectively.

We measured the gut microbial compositions of both the CVD no-risk and CVD risk groups. At the phyla level, the CVD risk group had a higher relative abundance of Proteobacteria (11.184 vs. 4.833%, *p* = 0.021) and lower relative abundances of Firmicutes (29.00 vs. 39.76%, *p* = 0.034) and Actinobacteria (0.91 vs. 2.81%, *p* < 0.014) than the CVD no-risk group (Figure 2A; Supplementary Figure 1). Although the CVD risk group had a higher proportion of Bacteroidetes (57.49%) than the CVD no-risk group (51.84%), statistically, the difference was not significant (Figure 2A; Supplementary Figure 1). We also estimated the Firmicutes and Bacteroidetes (F/B) and Firmicutes/Proteobacteria (F/P) ratios for the two groups. The CVD risk group F/B ratio showed no difference from that of the CVD no-risk group (*p* = 0.945) (Figure 2B), whereas the CVD risk group F/P ratio was significantly higher (49.4 vs. 10.13, *p* = 0.035) in the CVD risk group than that of the CVD no-risk group (49.4 vs. 10.13, *p* = 0.035) (Figure 2C).

We identified many genera that were significantly decreased in the CVD risk group compared with the CVD no-risk group, mainly the genera *Ruminococcus* (0.95 vs. 2.97%, *p* = 0.0002), unclassified Ruminocococcaceae family genera (5.82 vs. 10.36%, *p* = 0.014), and *Bifidobacterium* (0.75 vs. 2.20%, *p* = 0.03). Additionally, other genera, such as unclassified genera from the Mogibacteriaceae and Coriobacteriaceae families, and *Clostridium, Christensenella*, and *Lactobacillus*, were significantly lower in the CVD risk group than the CVD no-risk group (Figure 2D; Supplementary Figure 2).

A further exploration of the genus *Ruminococcus* to the species level identified *R. callidus* and an unclassified species from the Ruminococcaceae family as being significantly decreased in the CVD risk compared with the CVD no-risk group, whereas other species such as *R. bromii* and *R. flavefaciens* were not significantly changed. In addition, the relative abundances of other *Ruminococcus* species from the Lachnospiraceae family, such as *R. gnavus, R. torques*, and unclassified, were the same in the two groups (Figure 2E).

### Gut Microbial Diversity in Obese Subjects With Increased CVD Risk

**Figure 3:**
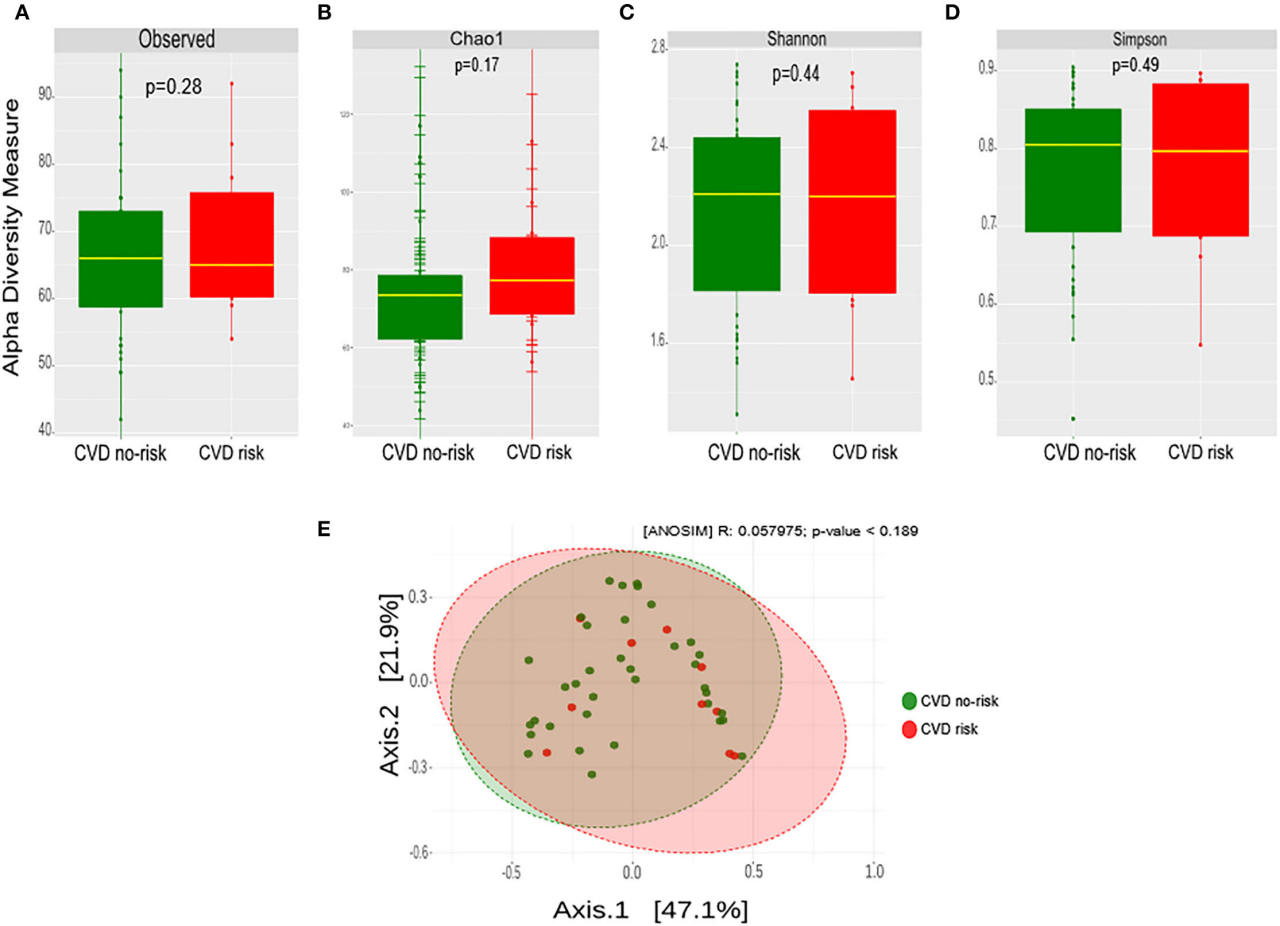
Gut microbial diversity in the CVD no-risk and CVD risk groups. **(A–D)** Alpha diversity was measured by the four commonly used methods, Observed, Chao1, Shannon, and Simpson. The boxplots show median and interquartile (IQR) range and whiskers extending to the most extreme points within 1.5-fold IQR. **(E)** Beta diversity index was measured by the Bray-Curtis method using principle coordinate analysis of the relative abundance of OTUs. The two-variances explained by Axis.1 and Axis.2 are 47.1 and 21.9%, respectively. ANOSIM, analysis of similarity; CVD no-risk, *N* = 36; CVD risk, *N* = 10. *P* < 0.05 was considered statistically significant using Student's *t-*test.

We estimated the microbial richness and abundance in the CVD risk and CVD no-risk groups using Observed, Chao1, Shannon, and Simpson methods. There was no significant change or shift in the microbial richness or the relative abundance in the CVD risk compared with the CVD no-risk group (Figures 3A–D). We estimated the similarities or dissimilarities between the two groups, and it did not uncover any similarities between the two groups (Figure 3E).

### Microbial Markers in Obese Subjects With Increased CVD Risk

**Figure 4:**
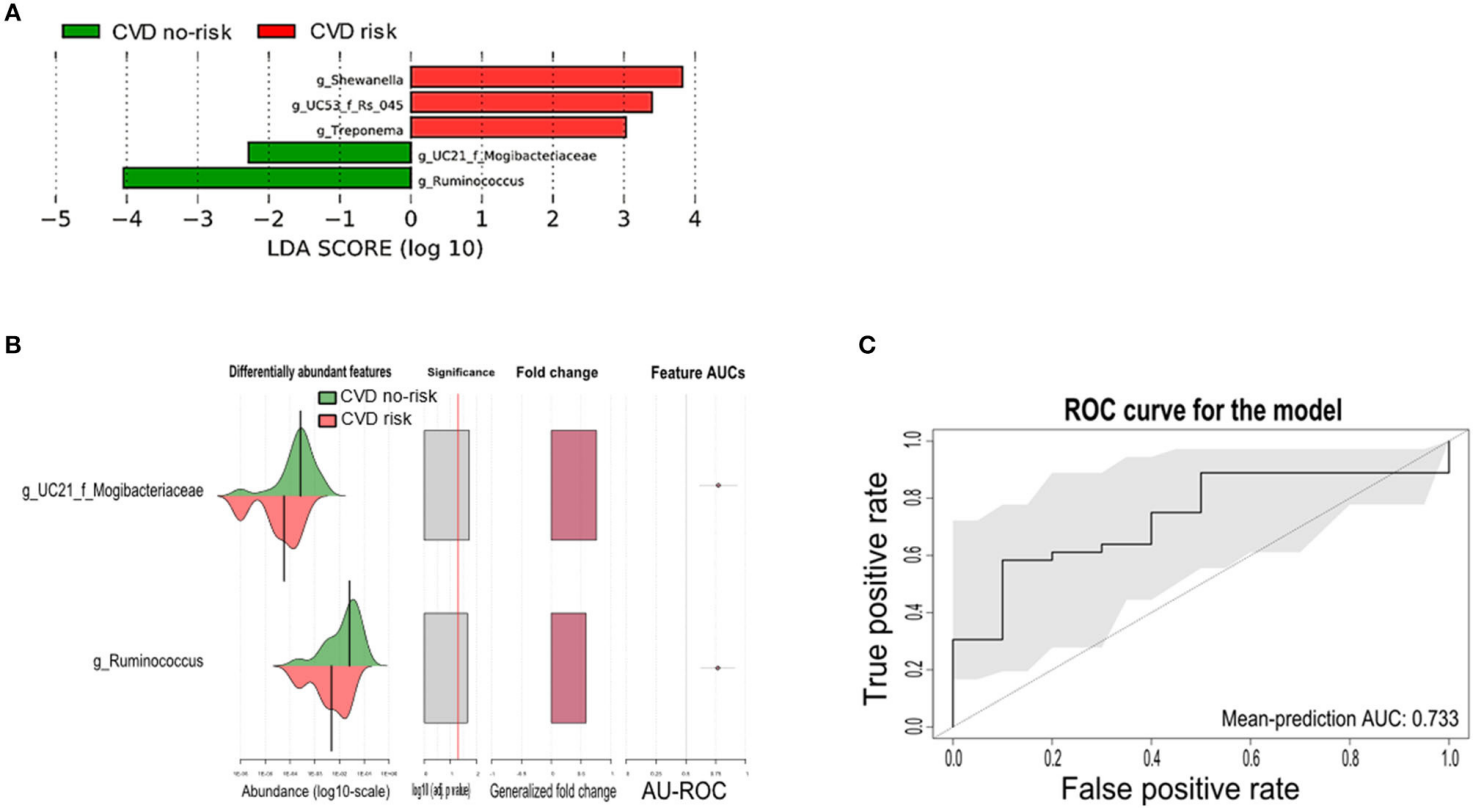
Gut microbial markers and their validation in the CVD risk group. **(A)** Gut microbial markers were measured by LEfSe analytical tool with an LDA cut-off value of >2.0 in both the CVD no-risk and CVD risk groups. **(B,C)** Gut microbial markers from LEfSe analysis were validated using the SIAMCAT tool, which displayed the cross-validation error as a receiver operating characteristic (ROC) curve with the 95% confidence interval shaded in gray. The area under the ROC (AUROC = 0.733) is given below the curve. The *x*-axis and *y*-axis represent false-positive and true-positive rates, respectively, for the tested markers. An AUROC value of more than 0.7 is considered fairly good in terms of the test's discriminative ability. CVD no-risk, *N* = 36; CVD risk, *N* = 10. “g_UC”: unclassified bacteria at the genus level.

Next, we performed LEfSe analysis to identify differential bacterial genera between the two groups. The genera *Ruminococcus, Shewanella, Treponema*, and unclassified genera from the Mogibacteriaceae and Rs_045 families were found to be microbial markers, of which, only the genus *Ruminococcus* and an unclassified genus from the Mogibacteriaceae family were present across all obese subjects, and these two genera were significantly enriched in the CVD no-risk group compared with the CVD risk group. The other bacterial genera, such as *Shewanella, Treponema*, and an unclassified genus from the Rs_045 family, were enriched in the CVD risk group, and interestingly, these were not present in all obese subjects (Figure 4A; Supplementary Figure 3).

We then evaluated the potential associations between the gut microbial markers and host phenotype using the SIAMCAT tool. We found that the gut microbial markers, such as the genus *Ruminococcus* and the unclassified genus from the Mogibacteriaceae family, were significantly enriched (mean AUC of the ROC curve was 0.733) and associated with the CVD no-risk group, suggesting their vital role in obesity-associated CVD (Figures 4B,C).

### Gut Microbiota-Modulated Metabolic Pathways That Are Associated With Increased CVD Risk in Obese Subjects

**Figure 5:**
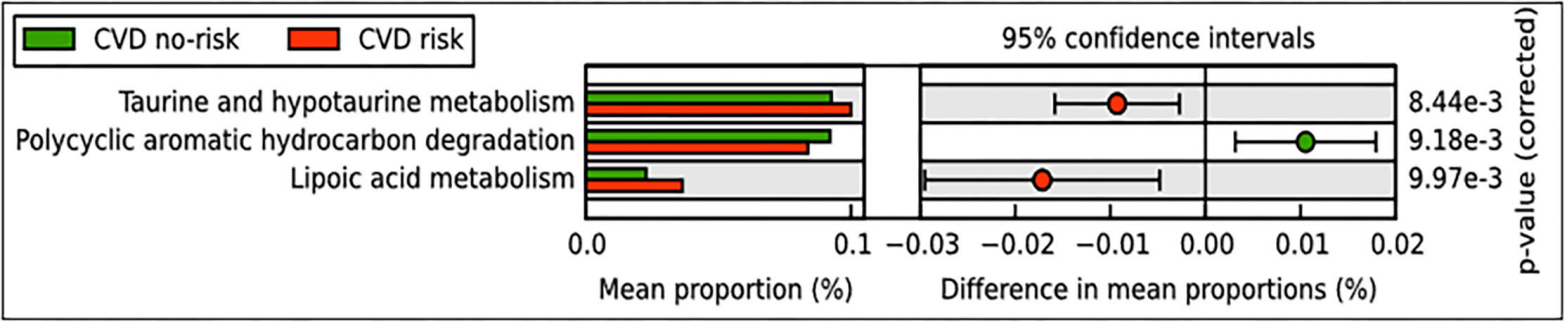
Predicted functional pathways in CVD no-risk and CVD risk groups. Predicted functional pathways were obtained using the PICRUSt method based on bacterial relative abundance shown as mean proportions in the CVD no-risk and CVD risk groups. *P* < 0.01 was considered statistically significant. CVD no-risk, *N* = 36; CVD risk, *N* = 10.

We performed PICRUSt analysis to find potential pathways that are modulated based on microbial abundance in the CVD risk and CVD no-risk groups. The results revealed that metabolism of lipoic acid, taurine, and hypotaurine pathways were elevated, whereas the polycyclic aromatic hydrocarbon degradation pathway was decreased in the CVD risk group compared with the CVD no-risk group (Figure 5).

### Nutrient Components Responsible for the Relative Abundance of Genus *Ruminococcus* in Obese Subjects

**Figure 6:**
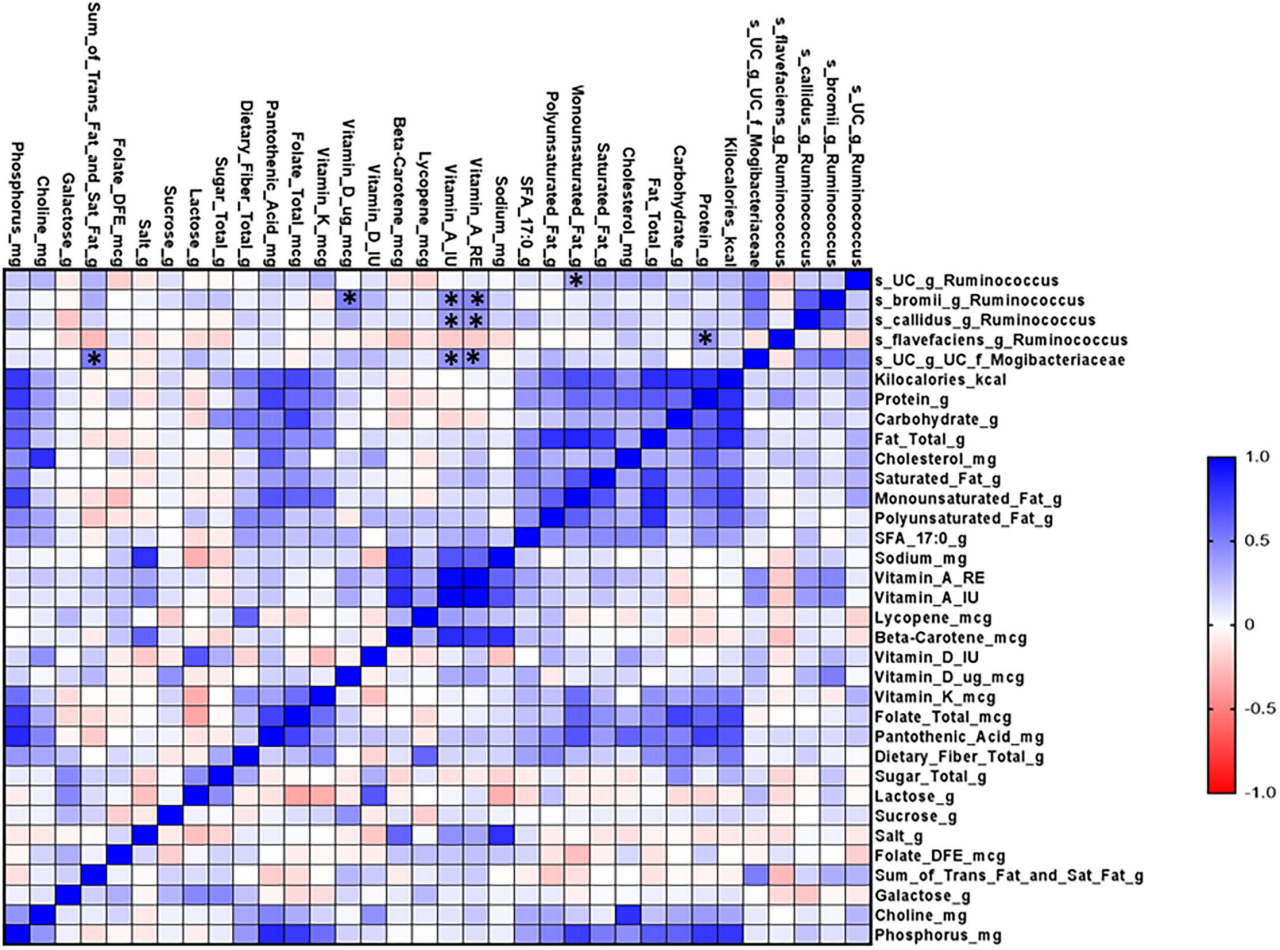
Microbial correlation matrix analysis of diet and gut microbiome in the CVD no-risk and CVD risk groups. Correlation matrix analysis was performed using GraphPad Prism software. The red line indicates a negative correlation and the blue line indicates a positive correlation. CVD no-risk, *N* = 30; CVD risk *N* = 8. *p < 0.05 was considered statistically significant using Student's *t-*test.

Finally, we performed correlation matrix analysis of data on diet and microbial composition. We found that different species of the genus *Ruminococcus* were positively correlated with the dietary intake of monounsaturated fat, vitamin D, vitamin A, and protein (Figure 6).

## Discussion

At the phyla level, three major bacteria were modulated in the CVD risk group in this study, namely Firmicutes (decreased), Proteobacteria (increased), and Actinobacteria (decreased), compared with those in the CVD no-risk group. Moreover, though the relative abundance of Bacteroidetes was higher in the CVD risk group than the CVD no-risk group, the difference was not statistically significant (*p* > 0.05) (Figure 2A). Interestingly, when we analyzed the F/B ratio, it was not significantly altered in the CVD risk group (Figure 2B). Also, Of note, Duncan et al. demonstrated that, in the clinical setting, Firmicutes and Bacteroidetes had no influence on obesity development (36). However, the reduction in the Firmicutes relative abundance was compensated by the increased relative abundance of Proteobacteria, rather than Bacteroidetes, in the CVD risk group, which is evident from the decreased Firmicutes to Proteobacteria (F/P) ratio (Figure 3C). Shirey et al. also reported no change in the F/B ratio, but they found a significant decrease in the F/P ratio in infant gut microbiota (37). Thus, changes in the abundance of Firmicutes compared with Proteobacteria could be more influential for obesity-associated CVD risk than the F/B ratio.

In our study, we found no change in microbial richness, relative abundance, or beta diversity between the CVD risk and CVD no-risk groups (Figures 3A–E), suggesting there was no major shift in microbial composition, but there were alterations in the relative abundances of a few bacteria that could play roles in the development of obesity-associated CVDs. The relative abundances of many genera were significantly reduced in the CVD risk group, especially *Ruminococcus, Bifidobacterium, Lactobacillus, Christensenella, Clostridium*, and unclassified genera from the Ruminococcaceae, Mogibacteriaceae, and Coriobacteriaceae families (Figure 2D; Supplementary Figure 2). Identification of microbial markers by LEfSe analysis revealed the CVD risk group was enriched in a few bacteria genera, such as *Shewanella, Treponema*, and an unclassified genus from the family Rs-045, and depleted in *Ruminococcus* and an unclassified genus from the Mogibacteriaceae family (Figure 4A). We also employed the SIAMCAT pipeline to understand the relationships between microbial communities and host phenotype and discovered that the genus *Ruminococcus* and an unclassified genus from the Mogibacteriaceae family were significantly associated with obesity-associated CVD risk (ROC curve AUC = 0.733) (Figures 4B,C).

Multiple studies have demonstrated that diet is one of the most prominent epigenetic factors affecting the gut microbial composition in terms of both bacterial richness and abundance (23, 24). It is believed that acute dietary adjustment can change the microbial composition dramatically, but it does not cause a switch from one enterotype to another due to its stability, whereas chronic dietary adjustment does facilitate enterotype switching (26). In addition to its stability, a classic feature of the enterotype is its reversibility. Enterotypes are affected by various factors, such as diet, lifestyle, age, and environmental stress (38, 39). In our study, 24-h dietary analysis revealed that the CVD risk group consumed lower amounts of MUFA, PUFA, vitamin D, total trans-fat and saturated fat, and phosphorus, and a higher amount of folate (Figure 1A).

The most common MUFAs in the diet are oleic acid, palmitoleic acid, vaccenic acid, and eicosenoic acid (40). Long-term high-fat consumption may significantly affect the host phenotype through the modulation of gut microbiota (Firmicutes, Bacteroidetes, Actinobacteria, and Proteobacteria) and gut microbiota-derived SCFAs, and monounsaturated fat (MUFA) reduces obesity and obesity-associated CVDs by activating the synthetic and catabolic pathways of triglyceride-rich lipoprotein metabolism (41, 42). Hidalgo et al. (43) demonstrated that supplementation with the MUFA (oleic acid) to spontaneously hypertensive rats for 12 weeks significantly improved their hypertension through the up-regulation of Ruminococcaceae. In the latest report, Goiri et al. described a positive correlation between the consumption of MUFA and the relative abundance of *Ruminococcus* in dairy cows (44). In this study, we found that intake of dietary MUFA was positively correlated with the relative abundance of *Ruminococcus*. A study conducted by Clarke and Mozaffarian suggested that the intake of MUFA (1% of total energy content) significantly altered various CVD risk factors, mainly complete lipid profiles and apolipoproteins (45). In addition, Mente et al. reported that MUFA intake negatively correlated with coronary heart disease (46). In contrast to this, several meta-analyses have suggested that MUFA intake does not affect CHD risk and related deaths (47, 48). Thus, it is unclear if MUFA intake has protective effects against CVDs, and more comprehensive intervention studies are needed to clarify these discrepancies. Overall, however, the intake of MUFA might prove have beneficial effects on CVD risk through the up-regulation of *Ruminococcus*. Furthermore, numerous studies have explained in detail how the intake of PUFAs, such as linolenic acid and linoleic acid, can protect against obesity-associated CVDs (49, 50). Noriega et al. (51) demonstrated that supplementation with omega-3 PUFAs for 2 weeks increased the relative abundance of the genus *Ruminococcus*. Interestingly, although the CVD risk group in our study consumed a lower amount of PUFAs, we did not find any correlation between PUFA and the relative abundance of the genus *Ruminococcus*, suggesting that the beneficial effects of PUFAs are mediated by alternative mechanisms other than modulation of the genus *Ruminococcus*.

Recent evidence suggests that the beneficial effects of vitamin D are mainly modulated via the gut microbiota, along with other demonstrated mechanisms (52–54). In this study, we found that vitamin D consumption was significantly lower in the CVD risk group than the CVD no-risk group. An interesting study recently conducted by Singh et al. (55) showed that vitamin D supplementation modulated the relative abundance of *Ruminococcus* in healthy subjects from Qatar. A point to be considered is that, when they categorized the subjects as responders (more than 20 ng/μl) or non-responders (<20 ng/μl) based on the dose of supplemented vitamin D, they found that higher vitamin D was related *Ruminococcus* bacteria species (*R. bromii*) enrichment, whereas when they compared data from subjects pre- or post-vitamin D supplementation, vitamin D supplementation inversely correlated with the relative abundance of the genus *Ruminococcus*. Furthermore, Thomas et al. (56) demonstrated that a higher level of 1,25(OH)_2_D (the active form of vitamin D) was positively associated with the genus *Ruminococcus*. Researchers should be careful in drawing conclusions on the roles of the genus *Ruminococcus* because its members are assigned to both Ruminococcaceae and Lachnospiraceae families and could have distinct biological roles related to vitamin D levels. In support of this, Ghaly et al. (57) demonstrated that vitamin D supplementation led to enrichment in both Ruminococcaceae and Lachnospiraceae families in a dose-dependent manner, i.e., moderate amounts of vitamin D increased Ruminococcaceae, whereas high amounts of vitamin D increased Lachnospiraceae, which can worsen colitis conditions. In our study, we found no change in the relative abundances of bacterial species from the Lachnospiraceae family, whereas species from Ruminococcaceae family (especially, *R. callidus* and an unclassified *Ruminococcus* species) decreased in the CVD risk group in comparison to the CVD no-risk group (Figure 2E). Furthermore, correlation analysis revealed that vitamin D intake was negatively correlated with obesity-associated CVD risk (Figure 1B); thus, vitamin D intake seems to be associated with the relative abundance of the genus *Ruminococcus*, and its levels are critical in defining the relative abundance of the genus *Ruminococcus* from either Ruminococcaceae or Lachnospiraceae families.

*Ruminococcus* is a member of one of the three enterotypes (Enterotype 3) that are thought to be involved in shaping core microbiota; enterotypes are not often perturbated by acute stimuli, rather are believed to be stable (58). Contrary to this, Liang et al. (59) found another enterotype dominated by Enterobacteriaceae in a population in Taiwan, suggesting that enterotypes are quite robust among populations. *Ruminococcus* has a predominant role in the fermentation of dietary fiber to produce SCFAs, butyrates that are important energy sources for intestinal epithelial cells (60). Butyrates can have beneficial actions, such as maintaining intestinal barrier function and regulating the expression of various genes related to lipids, inflammatory and immune responses, and cell cycle processes (61–63) through complex mechanisms, such as inhibiting histone deacetylation, secreting glucagon-like peptide 1, regulating inflammatory mediators, enhancing antioxidant capacity, and activating the PPAR-γ pathway (64–66). Many studies have suggested that a reduction in butyrate production can harm the homeostasis of gut flora and cause leaky gut, which has been linked to the development of obesity-associated CVDs (67). Interestingly, when we analyzed the dietary data, we did not find any difference in dietary fiber intake between the CVD risk and CVD no-risk groups (Figure 1A), suggesting that the genus *Ruminoccoccus* has a beneficial role other than butyrate production. In addition, contrary reports suggest that age can influence the relative abundance of *Ruminococcus*, which decreases with age (68), whereas Arumugam et al. reported that *Ruminococcus* are not modulated by age, BMI, gender, or nationality (58), rather it was demonstrated to be modulated according to dietary constituents (69). In this study, we also found that subjects in the CVD risk group had a significantly higher median age than the CVD no-risk group (Table 1). Therefore, because of its heterogenicity, there may be both positive and negative effects from *Ruminococcus*; some species (for example *R. bromii*) has shown potential beneficial effects through degradation of resistant starch in shaping the gut microbial composition (70), whereas *R. gnavus* has been implicated in the pro-inflammatory process in Crohn's disease (71). In this study, we identified many species of the genus *Ruminococcus* from the Ruminococcaceae family and found *R. callidus* and unclassified *Ruminococcus* species to be significantly decreased in the CVD risk group. In addition, other *Ruminococcus* species of the Lachnospiraceae family were unaltered in the CVD risk group, which potentially explains the heterogeneous role of the genus *Ruminococcus*.

Obesity is an important risk factor for CVDs and promotes disease development through complex mechanisms; predominantly through the activation of inflammatory and oxidative stress pathways (10, 72). Obesity is also a key factor in the development of liver injury, insulin resistance, and diabetes mellitus. Li et al. (73) reported that the relative abundance of the genus *Ruminococcus* was lower in non-alcoholic fatty liver disease (NAFLD) patients compared with healthy subjects, but Boursier et al. (74) and others found that *Ruminococcus* sp. were decreased in patients with fibrosis. Lee et al. (75) also found that *Ruminococcus* species, especially *R. faecis*, reduced the increased levels of ALT seen in an experimental NAFLD mouse model. In our study, the CVD risk subjects also had higher levels of ALT and diabetic parameters such as HbA1c and FBG than the CVD no-risk subjects (Table 1). Pathway analysis by the PICRUSt tool revealed many pathways that were modulated, especially taurine and hypotaurine metabolism, lipoic acid metabolism, and the polycyclic aromatic hydrocarbon degradation pathway (Figure 5). Taurine and hypotaurine are non-essential amino acids that can be obtained from the diet. The heart, skeletal muscle, and retina were found to have high concentrations of taurine (76, 77), and taurine has been involved in many diverse biological functions, primarily bile salt formation and fat digestion. The primary roles of taurine are its antioxidant and anti-inflammatory properties. A handful of clinical trials on taurine supplementation have suggested that taurine significantly improves many pathological conditions, including dilated cardiomyopathy, ischemic heart disease, hypertension, congestive heart failure, atherosclerosis, and obesity (78, 79). In addition, Fang et al. demonstrated that taurine supplementation significantly ameliorated the reduction in the relative abundance of *Ruminococcus* in immunosuppressed mice, suggesting a positive correlation between these two entities (80). Moreover, lipoic acid, a potent natural antioxidant, has been reported to alleviate various CVDs associated with elevated levels of oxidative stress (81). Polyaromatic hydrocarbons are known carcinogens and have been implicated in the pathogenesis of various CVDs through the activation of inflammatory responses (82, 83). In our study, we found that the CVD risk group had higher levels of taurine, hypotaurine, and lipoic acid metabolism and lower levels of polycyclic aromatic hydrocarbon degradation than the CVD no-risk group (Figure 5). Thus, it seems that the genus *Ruminococcus* and an unclassified genus of the Mogibacteriaceae family might prevent obesity-associated CVDs through the suppression of oxidative stress and inflammatory responses.

Out pilot study had some limitations, such as (1) the CVD risk group included fewer subjects than the CVD no-risk group and (2) recording of food intake was based on the 24-h recall method. However, the findings from this pilot study are promising and will be further evaluated in a comprehensive study with a larger cohort.

## Conclusions

**Figure 7:**
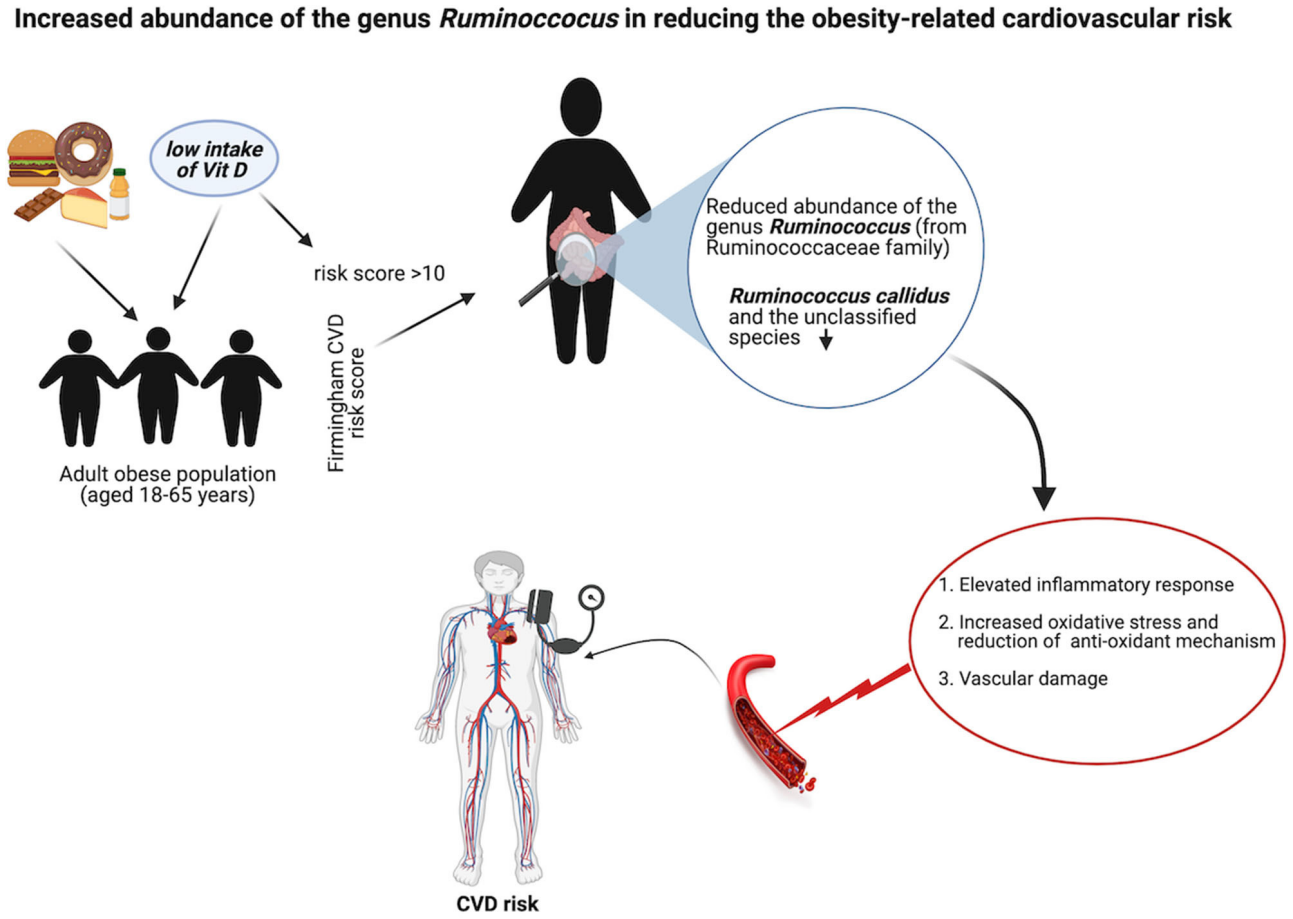
Schematic representation of the role of Ruminococcus in obese-related CVD. Created with BioRender.com.

Although there were fewer samples in the CVD risk group, the findings clearly showed this group had gut microbial dysbiosis that did not cause a drastic change in the gut microbial diversity but rather a moderate shift from Firmicutes to Proteobacteria. This minor shift had a potentially negative effect on obesity severity. The reduced relative abundance of *Ruminococcus* from the Ruminococcaceae family and an unclassified genus from the Mogibacteriaceae family significantly increased the risk of CVDs, and this unfavorable effect might be mediated by increased oxidative stress and the activation of inflammatory pathways. However, future mechanistic and intervention studies are warranted to confirm the findings of this study, which could be useful for the management of CVDs in obese conditions. We summarized this hypothesis in the graphical abstract (Figure 7).

## Data Availability Statement

The datasets presented in this study can be found in online repositories. The names of the repository/repositories and accession number(s) can be found below: https://www.ncbi.nlm.nih.gov/, PRJNA785127.

## Ethics Statement

The study participants were approached and recruited after having consented at Hamad Medical Corporation, Doha, Qatar. This study was approved by the Institutional Review Board from both the Sidra Medicine (IRB, #1604002867) and Hamad Medical Corporation (IRB, #16419/16).

## Author Contributions

AT and WE designed the study and reviewed the manuscript. WE was involved in the subject recruitment process and sample collection process. AL and DB processed the samples. AL performed gut microbiome data analysis and wrote the manuscript. SA-S performed diet assessment. SA analyzed dietary intake. All authors contributed to the article and approved the submitted version.

## Funding

This study was supported by Sidra Medicine, Doha, Qatar (SDR400005).

## Conflict of Interest

The authors declare that the research was conducted in the absence of any commercial or financial relationships that could be construed as a potential conflict of interest.

## Publisher's Note

All claims expressed in this article are solely those of the authors and do not necessarily represent those of their affiliated organizations, or those of the publisher, the editors and the reviewers. Any product that may be evaluated in this article, or claim that may be made by its manufacturer, is not guaranteed or endorsed by the publisher.

## Abbreviations

ALP: alkaline phosphatase
ALT: alanine transaminase
AST: aspartate transaminase
BMI: body mass index
BMR: basal metabolic rate
BMR_C: basal metabolic rate calorie
BP: blood pressure
CVDs: cardiovascular diseases
F/B: Firmicutes/Bacteroidetes
F/P: Firmicutes/Proteobacteria
FBG: fasting blood glucose
FFM: free fat mass
FM: fat mass
HbA1c: glycosylated hemoglobin
HDL: high-density lipoprotein
HR: heart rate
HT: height
LDL: low-density lipoprotein
MM: muscle mass
MUFA: monounsaturated fat
PUFA: polyunsaturated fat
SCFAs: short-chain fatty acids
T3: triiodothyronine
T4: thyroxine
TC: total cholesterol
TG: triglycerides
TMAO: trimethylamine-N-oxide
TSH: thyroid-stimulating hormone.
