# Supplementary material for: Increased Relative Abundance of Ruminoccocus Is Associated With Reduced Cardiovascular Risk in an Obese Population
Source: Front Nutr. 2022 Apr 28;9:849005. doi: 10.3389/fnut.2022.849005 (PMC9097523; doi:10.3389/fnut.2022.849005)

Supplementary Table 1. Number of study participants for the profiling of the clinical and biochemical parameters in the CVD no-risk and CVD risk groups.

	Number of study participants	
	**CVD no-risk**	**CVD risk**
Clinical parameters		
Heart rate (HR)	33	10
Systolic BP	35	10
Diastolic BP	36	10
Diabetes mellitus-related parameters		
Fasting blood glucose (FBG)	31	10
HbA1c	32	10
Insulin	30	8
Complete lipid profiles		
Total cholesterol (TC)	31	10
Triglycerides (TG)	31	10
High-density lipoprotein (HDL)	31	10
Low-density lipoprotein (LDL)	31	10
Liver function parameters		
Alkaline phosphatase (ALP)	33	9
Alanine transaminase (ALT)	33	9
Aspartate transaminase (AST)	33	9
Thyroid function parameters		
Thyroid-stimulating hormone (TSH)	32	9
Triiodothyronine (T3)	27	7
Thyroxine (T4)	29	8
Vitamins		
Folate (vitamin B9)	31	8
Vitamin D	33	10
Vitamin B12	32	10
Uric acid	28	8
Iron saturation level	30	10

Supplementary Figure 1. Gut microbial composition (phyla level) in the CVD no-risk and CVD risk groups

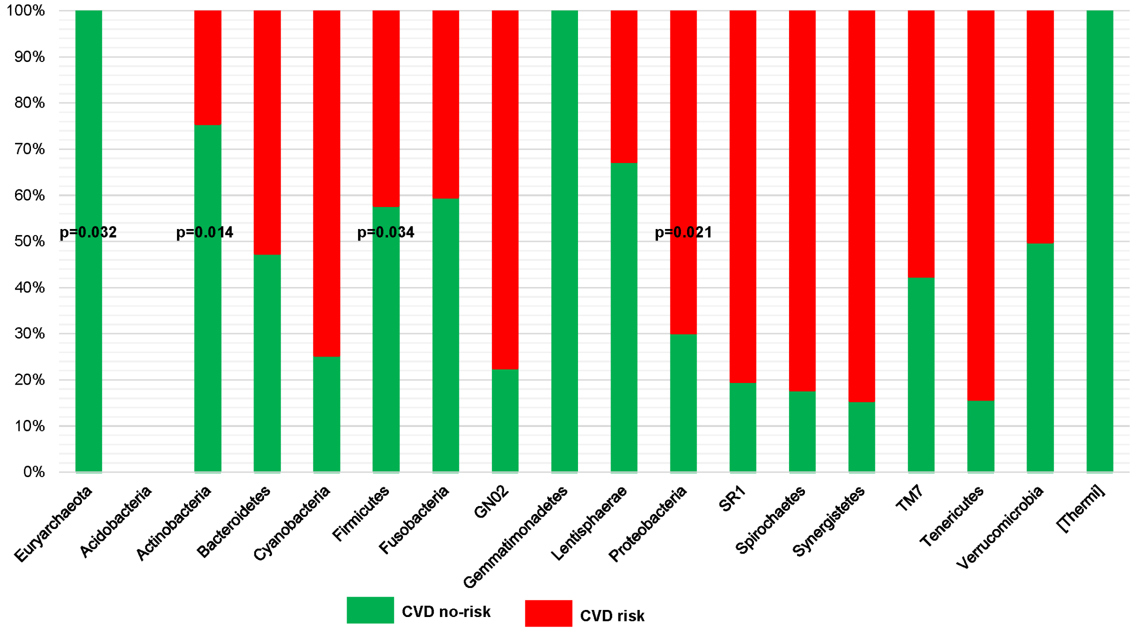

Supplementary Figure 2. Gut microbial composition (genus level) in the CVD no-risk and CVD risk groups

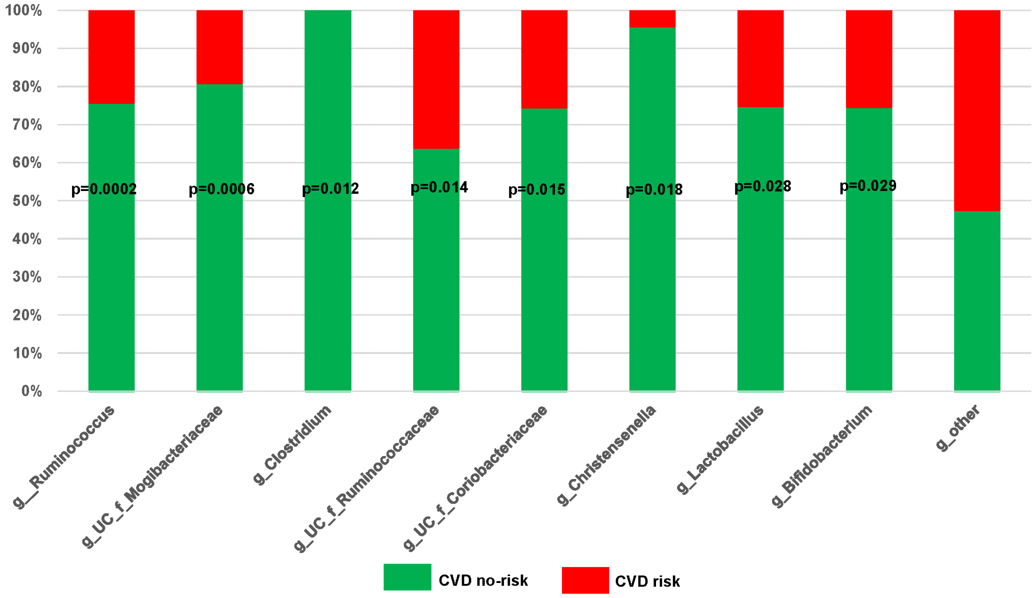

Supplementary Figure 3. Distribution of genera *Ruminococcus*, *Shewanella*, *Treponema*, and unclassified genera from the Mogibacteriaceae and Rs_045 families in the CVD no-risk and CVD risk subjects

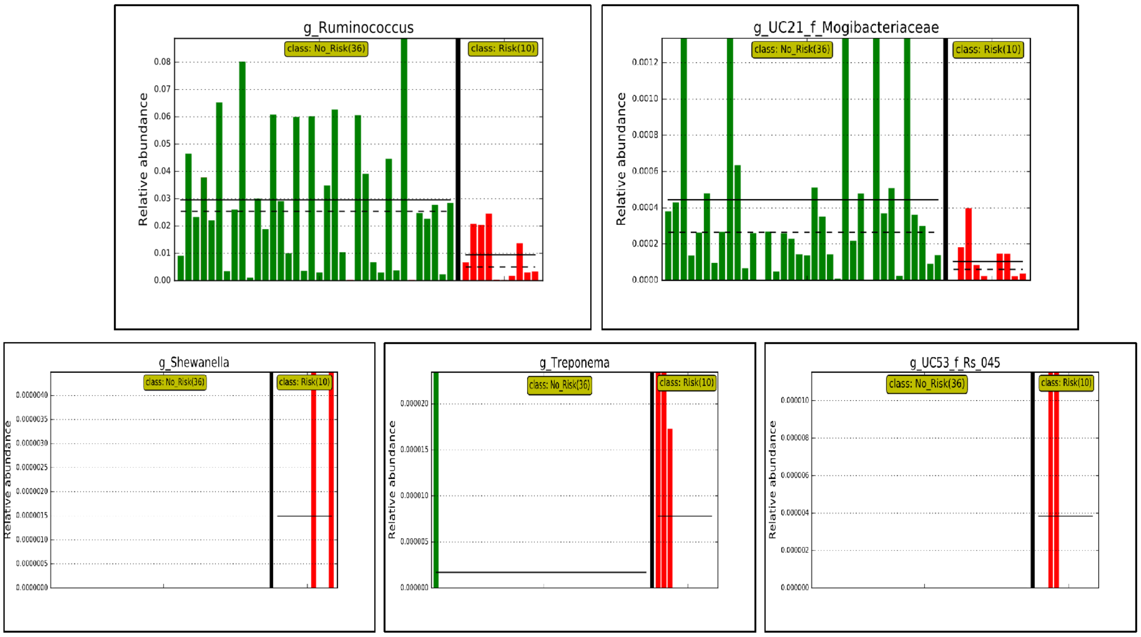

Supplement: Supplementary file 1 [file Data_Sheet_1.PDF]
